# Segregation of Neural Circuits Involved in Social Gaze and Non-Social Arrow Cues: Evidence from an Activation Likelihood Estimation Meta-Analysis

**DOI:** 10.1007/s11065-023-09593-4

**Published:** 2023-04-17

**Authors:** Claudia Salera, Maddalena Boccia, Anna Pecchinenda

**Affiliations:** 1 Ph.D. Program in Behavioural Neuroscience, Department of Psychology, “Sapienza” University of Rome, Via Dei Marsi, 78, 00185 Rome, Italy; 2 Department of Psychology, Sapienza University of Rome, Via Dei Marsi, 78, 00185 Rome, Italy; 3 Cognitive and Motor Rehabilitation and Neuroimaging Unit, IRCCS Santa Lucia, Rome, Italy

**Keywords:** Gaze, Arrow, Frontal gyrus, Temporoparietal junction, Superior temporal sulcus, ALE meta-analysis

## Abstract

Orienting attention by social gaze cues shares some characteristics with orienting attention by non-social arrow cues, but it is unclear whether they rely on similar neural mechanisms. The present ALE-meta-analysis assessed the pattern of brain activation reported in 40 single experiments (18 with arrows, 22 with gaze), with a total number of 806 participants. Our findings show that the network for orienting attention by social gaze and by non-social arrow cues is in part functionally segregated. Orienting by both types of cues relies on the activity of brain regions involved in endogenous attention (the superior frontal gyrus). Importantly, only orienting by gaze cues was also associated with the activity of brain regions involved in exogenous attention (medial frontal gyrus), processing gaze, and mental state attribution (superior temporal sulcus, temporoparietal junction).

## Introduction

The ability to shift attention based on the direction of eye gaze of another person is at the core of social attention (e.g., Baron-Cohen, 1995; Frith & Frith, 2001) and it has been extensively investigated using a variant of the standard attentional cueing paradigm (Posner, 1980). In this variant, the central symbolic cue is replaced by a face gazing left or right, and participants respond as quickly as possible to a peripheral target presented shortly after the gaze cue (Friesen & Kingstone, 1998). When gaze direction is not informative of where the target appears (i.e., 50% cue validity), a typical pattern of faster responses to targets presented at the spatial location looked at by the face (i.e., valid, or congruent cue) compared to targets presented at the opposite spatial location (i.e., invalid or incongruent cue) is observed (see McKay et al., 2021, for a recent review of gaze cueing effects). Because this effect is fast, as it occurs within 200 ms from the cues, and because it is observed with non-informative and even counter-informative central cues, it has been taken to indicate that it relies on exogenous mechanisms for orienting attention. This is because to elicit endogenous shifts of attention with purely symbolic cues, such as when a central stimulus characteristic is arbitrarily associated with a spatial location (e.g., a yellow circle indicates left and a blue circle indicates right), the cue needs to be predictive of target location and the SOA needs to be longer (> 300 ms) to engender cueing (e.g., Funes et al., 2007; Dodd & Wilson, 2009). Considering the social and biological relevance of faces, it had been originally proposed that orienting to eye gaze represents a unique attentional process that is qualitatively distinct from orienting based on other symbolic, central cues (e.g., Langton & Bruce, 1999; Driver et al., 1999; Friesen & Kingstone, 1998). However, this proposal is challenged by evidence of similar cueing effects observed with central, non-social cues such as arrow-cues (Hommel et al., 2001; Ristic et al., 2002; Tipples, 2002). Therefore, that gaze and arrow central cues shift attention even when they are not predictive of target location, with short SOAs suggesting that these cues may involve shifting attention that shares some characteristics of reflexive orienting typically observed with peripheral cues. Importantly, arrow and gaze also produce cueing effects that are greater than the summed effects of reflexive and volitional orienting. Accordingly, these effects have been attributed to gaze and arrows being perceptually asymmetrical signals or being overlearned directional signals, not requiring interpretation, which would challenge a dichotomous distinction in exogenous and endogenous attention. Indeed, in a recent review, Dalmaso et al. (2020) highlights that gaze and arrow cues may be associated with different forms of automatic orienting of attention.

Although the mechanisms underlying gaze and arrow cueing effects on attention have been extensively investigated (see Chica et al., 2014 for a review), it is still debated to what extent cueing effects elicited by central gaze and arrow cues rely on similar neural underpinnings. On one hand, it has been proposed that orienting attention based on gaze and arrow cues is qualitatively similar as both rely upon the dorsal and ventral frontoparietal networks, and the only difference is quantitative as orienting by gaze cues involves the lateral occipital regions (e.g., Tipper et al., 2008). Indeed, frontoparietal brain networks play an important role in spatial attentional orienting, and the debate is on whether there is a single attentional system supporting both endogenous and exogenous attention, or two anatomically and functionally different attentional systems (for a review see Chica et al., 2014). For instance, Corbetta et al. (2008) put forward the hypothesis of a bilateral, dorsal frontoparietal network for orienting both endogenous and exogenous attention and of a right-lateralized, ventral frontoparietal counterpart for reorienting to task-relevant events. The dorsal attention system is associated with covert and overt shifts of spatial attention, eye movements, and hand-eye coordination (Corbetta & Shulman, 2002) and includes regions in the frontal eye fields, ventral premotor cortex, superior parietal lobule, intraparietal sulcus, and motion-sensitive middle temporal area (MT+). Recent findings from fMRI studies show positive correlations between the activity of these regions (Laufs et al., 2003; Fox et al., 2005, 2006; Vincent et al., 2006). On the other hand, a key difference between gaze and arrows is that only orienting attention based on observed gaze direction is taken to indicate that we spontaneously attribute a mental state to others as we understand that a person looks at what they are interested in (see Senju et al., 2004; Pecchinenda & Petrucci, 2021). Therefore, the neural mechanisms involved in orienting attention by gaze cues should rely also on brain areas involved in mental state attribution and Theory of Mind (e.g., Calder et al., 2002) – that is the human ability to ascribe mental states to others. In contrast, the neural mechanisms involved in orienting attention based on arrow cues should not. Indeed, a large network of brain regions has been associated with mental state attribution and social cognition, including the medial prefrontal cortex, posterior superior temporal sulcus, temporoparietal junction (Caruana et al., 2015; Redcay et al., 2012), intraparietal sulcus (Saito et al., 2010), occipital gyrus (Oberwelland et al., 2016), precuneus, insula, and amygdala (Caruana et al., 2015).

In sum, orienting by gaze and arrow cues shares some characteristics of endogenous (i.e., it occurs with central cues) and some of exogenous (i.e., short SOA, it occurs even with non-predictive cues) cueing, but the question of whether orienting by social and non-social cues differs regarding the neural mechanisms involved is still unresolved. However, this can be addressed by performing a coordinate-based Activation Likelihood Estimation (ALE) meta-analysis on the available fMRI studies as it provides a synthesis of previous results, and it allows resolving conflicting views while overcoming the limitations of single studies (e.g., small sample size, low power, and generalization). Importantly, as we are interested in higher order processes (i.e., orienting by gaze and arrow cues) and to maximise the number of studies that could be included in the ALE meta-analysis, we are not distinguishing between predictive and non-predictive cues. That is, for both gaze and arrow cues, we are considering converging evidence about the brain activation maps when gaze and arrow cues are predictive as well as when they are non-predictive of target location. This strategy is in line with recommendations by Muller et al. (2018). Albeit meta-analyses of the neural networks involved in social attention are available, to our knowledge a direct comparison of the neural substrates of orienting attention based on gaze and arrow cues is missing.

To this aim, we conducted an ALE-meta-analysis with the following main purposes: (1) to provide a synthesis of the main brain networks involved in gaze and arrow cueing; (2) to test the hypothesis that different neural networks underlie orienting attention based on gaze and arrow cues. Even if this latter issue has been investigated using behavioural and EEG measures (e.g., Ristic et al., 2002, Hietanen et al., 2008; Brignani et al., 2009), no coordinate-based meta-analysis has so far been performed.

## Meta-Analysis

### Selection Criteria

We conducted a systematic review of the literature according to the Preferred Reporting Items for Systematic Reviews and Meta-Analyses, (PRISMA, Page et al., 2021; see Appendix Table 5 for the PRISMA Checklist on how PRISMA guidelines were followed). We searched the literature using different databases (i.e., PubMed, Scopus, and Web of Science) for articles published up to December 2021 using the following search syntax: (a) “fMRI” AND “attention” AND “arrow” (PubMed: 48; Scopus: 1072, Web of Science: 46); (b) “fMRI” AND “attention” AND “gaze” (PubMed: 214; Scopus: 258; Web of Science: 218). The search was performed on whole articles published in English. In total, 1856 potential articles were found (PubMed: 262; Scopus: 1330; Web of Science: 264). After duplicates were removed (N = 257), 1599 articles were screened according to the following a priori criteria:

Inclusion criteria:

1. Articles reporting whole-brain analyses performed using functional magnetic resonance imaging (fMRI);
2. Articles in which coordinates of activation foci were provided either in Montreal Neurological Institute (MNI) or in Talairach reference space;
3. fMRI studies with a visuo-perceptual/motor control condition to exclude all activations not directly related to visual orienting;
4. Studies with non-pathological groups;
5. Studies involving attentional orienting by central cues;
6. Group studies.

Exclusion criteria:

1. Studies reporting only results from region of interest (ROI) analyses;
2. Studies reporting only results of multivariate analyses (e.g., representational similarity or decoding analyses) or connectivity analyses (e.g., psychophysiological interaction analyses or dynamic causal modelling);
3. Studies involving pharmacological manipulations, psychotherapeutic interventions, or other manipulations of the participants’ psychophysical conditions;
4. Studies using emotional stimuli and auditory cues to orient attention;
5. Reviews, book chapters, books, conference papers, and meta-analyses;
6. Single case reports;
7. Studies contrasting experimental conditions with a low-level baseline (e.g., rest condition);
8. Studies in which arrow and gaze stimuli were used only for passive viewing;
9. Studies using a judgement task on cue direction;
10. Studies not reporting contrasts specific for each type of cue (i.e., arrow, gaze).

**Fig. 1:**
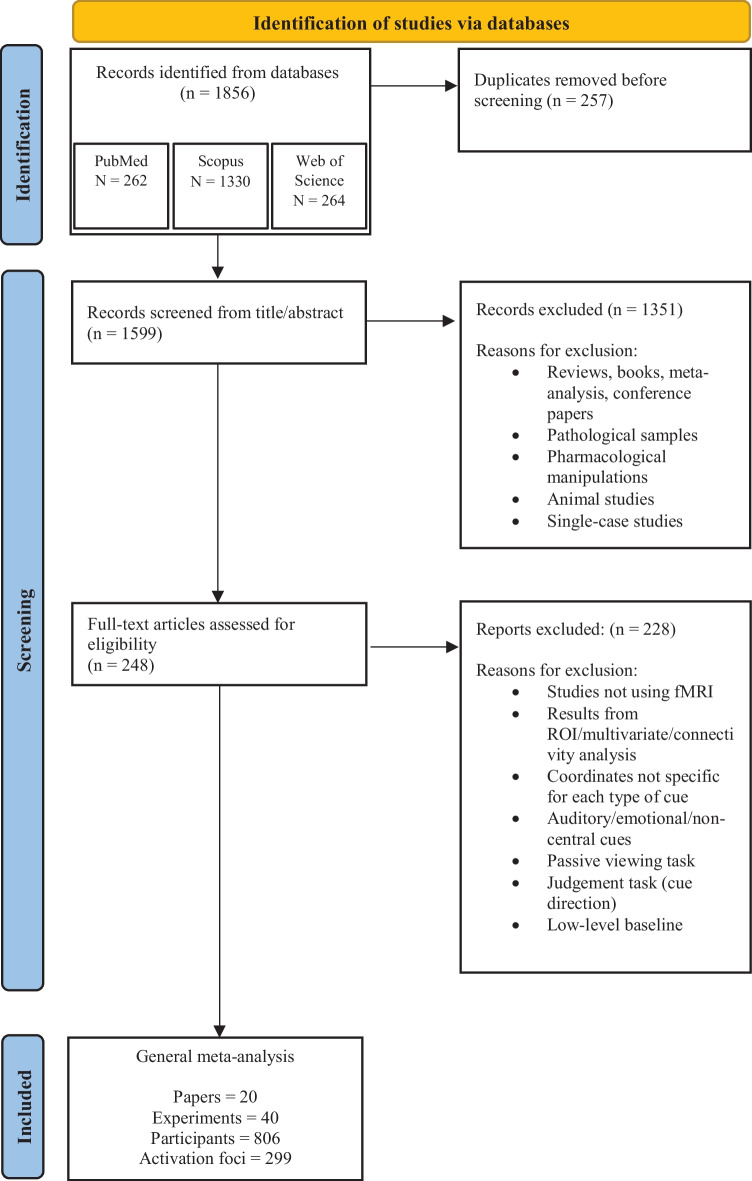
PRISMA workflow chart illustrating relevant details about literature selection procedures and materials included in the meta-analysis

Accordingly, 248 articles were selected to be assessed for eligibility. After examining the whole content, 20 articles satisfied the adopted criteria and were included in the ALE meta-analysis (see Fig. 1). One investigator conducted the search literature, performed the removal of duplicates, and selected articles based on inclusion criteria. The other investigators double-checked the final selection.

The meta-analysis was performed on 40 single experiments, involving a total number of 806 participants, extracted from the 20 selected papers. All experiments included contrasting attentional orienting to a high-level control condition (e.g., word-related processing, visuo-perceptual processing), to assess that all activations were directly related to the central spatial cueing. There were 18 experiments with arrow cues from 11 papers, and 22 experiments with gaze cues from 11 papers (in two papers, both arrow and gaze cues were used).

Table 1 provides the complete list of contrasts included in the meta-analysis, along with details about (1) the paper from which they were extracted; (2) the ALE analysis in which they were included; (3) the type of cue used.

**Table 1:** Studies included in the ALE meta-analysis

**Article**	**Task**	**Sample Size**	**N° of contrasts**	**Contrast**	**ALE** **meta - analysis**	**Cue type**	**Predictive Validity**
Böckler et al. (2016)	Discrimination task (gaze cueing task)	21	6	Eye contact > averted gaze	Social	Gaze	NP (50%)
				Averted gaze > eye contact	Social		
				Congruent gaze cues > incongruent gaze cues	Social		
				Incongruent gaze cues > congruent gaze cues	Social		
				Eye contact congruent > averted gaze congruent	Social		
				Averted gaze incongruent > averted gaze congruent	Social		
Caruana et al. (2015)	Target localization task (joint attention task)	13	2	Responding to joint attention (RJA > RJAc)	Social	Gaze	P (100%)
				Conjunction of initiating and responding to joint attention	Social		
Dombert et al. (2016)	Discrimination task (arrow cueing task)	24	1	Spatial reorienting: invalid > valid	Non-social	Arrow/Feature	P (70–90%)
Greene et al. (2009)	Discrimination task (gaze cueing task)	20	1	Gaze cue > square cue	Social	Gaze/Square	NP (50%)
Hietanen et al. (2006)	Detection task (gaze/arrow cueing task)	10	2	Directional > non directional cuing by gaze cues	Social	Gaze/Arrow	NP (50%)
				Directional > non directional cuing by arrow cues	Non-social		
Joseph et al. (2014)	Discrimination task (gaze/arrow cueing task)	20	5	Gaze reorienting	Social	Gaze/Arrow	NP (50%)
				Gaze > arrow reorienting, invalid > valid	Social		
				Gaze cueing correlation with RTs facilitation	Social		
				Arrow orienting	Non-social		
				Arrow > gaze reorienting, invalid > valid	Non-social		
Koike et al. (2019)	Target localization task (joint attention task)	65	1	Main effect of JA [IJA + dIJA + RJA + dRJA − 4xCTRL]	Social	Gaze	NP (50%)
Lee et al. (2010) (Exp. 1)	Detection task (gaze-head cueing task)	17	2	Turning heads > moving scrambles	Social	Gaze-Head orientation	NP (50%)
				Turning heads > static heads	Social		
Mao et al. (2007)	Detection task (arrow cueing task)	12	2	Rvf > fixation	Non-social	Arrow	NP (50%)
				Lvf > fixation	Non-social		
Natale et al. (2009)	Discrimination task (arrow cueing task)	22	2	Orienting of endogenous spatial attention (valid > neutral trials)	Non-social	Arrow/Peripheral cue	P (75%)
				Spatial reorienting following endogenous invalid cues (invalid > valid trials)	Non-social		
Noesselt et al. (2002)	Discrimination task (arrow cueing task)	6	2	Attention right > left	Non-social	Arrow	NP (50%)
				Attention left > right	Non-social		
Peelen et al. (2004)	Discrimination task (arrow/exogenous cueing task)	19	1	Cue > neutral	Non-social	Arrow	NP (50%)
Redcay et al. (2010) (Exp. 2)	Target localization task (joint attention task)	13	1	Joint attention condition (JA) > solo attention (SA)	Social	Gaze/Object	P (100%)
Redcay et al. (2012)	Target localization task (joint attention task)	32	2	Responding to joint attention (RJA) > solo attention (SA)	Social	Gaze/Object	P (100%)
				Responding to joint attention (RJA) > initiating joint attention (IJA)	Social		
Sato et al. (2016)	Discrimination task (gaze cueing task)	27	1	averted > straight gaze (supraliminal only)	Social	Gaze	NP(50%)
Small et al. (2003)	Discrimination task (arrow cueing task)	15	3	(V + trials) > (ND trials)	Non-social	Arrow	P (80%)
				(V + trials) > (V - trials)	Non-social		
				(V-trials) > (V + trials)	Non-social		
Steinkamp et al. (2020)	Discrimination task (arrow cueing task)	27	2	Invalid > valid horizontal	Non-social	Arrow	P (80%)
				Invalid > valid vertical	Non-social		
Thiel et al. (2004)	Detection task (arrow cueing task)	13	1	reorienting (i.e., invalid > valid trials)	Non-social	Arrow	P (80%)
Turk-Browne et al. (2013)	Detection task (gaze cueing task)	31	2	Facilitation effects, Cued-unsure > uncued trials	Social	Gaze	P (80%)
				Reorienting effects, Uncued > cued-unsure trials	Social		
Weissman & Prado (2012)	Discrimination task (arrow cueing task)	14	1	Greater activity in invalid than in valid trials	Non-social	Arrow	P (75%)

For each article, the table provides details about tasks, number of participants, number and label of contrasts, classification of each contrast in the ALE analysis (Social, Non-social), cue type, and cue predictive validity (P = predictive cue; NP = non-predictive cue)

Importantly, although all included experiments used similar methodologies, they also differed in some aspects. More specifically, in some experiments, only one type of cue was used (e.g., arrow cues: Dombert et al., 2016; Mao et al., 2007; Natale et al., 2009; Noesselt et al., 2002; Peelen et al., 2004; Small et al., 2003; Steinkamp et al., 2020; Thiel et al., 2004; Weissman & Prado, 2012; gaze cues: Böckler et al., 2016; Caruana et al., 2015; Koike et al., 2019; Lee et al., 2010; Sato et al., 2016; Turk-Browne et al., 2013). In contrast, in other experiments, both social and non-social cues were used (e.g., Greene et al., 2009; Hietanen et al., 2006; Joseph et al., 2014; Redcay et al., 2010, 2012). While experiments with non-social cues, or with both arrow and gaze cues, involved similar tasks (i.e., detection tasks, e.g., Hietanen et al., 2006; Mao et al., 2007; Thiel et al., 2004; discrimination tasks, e.g., Dombert et al., 2016; Greene et al., 2009; Joseph et al., 2014; Natale et al., 2009; Noesselt et al., 2002; Peelen et al., 2004; Small et al., 2003; Steinkamp et al., 2020; Weissman & Prado, 2012), experiments with social cues could either involve detection or discrimination tasks (e.g., Böckler et al., 2016; Lee et al., 2010; Sato et al., 2016; Turk-Browne et al., 2013), or joint attention tasks (e.g., Caruana et al., 2015; Koike et al., 2019, 2010; Redcay et al., 2012). In this latter case, to allow comparisons with the typical gaze cueing tasks, only joint attention studies that required detection or discrimination of targets presented at the cued/uncued location were included. Finally, the included experiments differed in terms of cue predictive validity as cues could be predictive (e.g., Caruana et al., 2015; Dombert et al., 2016; Natale et al., 2009; Redcay et al., 2010, 2012; Small et al., 2003; Steinkamp et al., 2020; Thiel et al., 2004; Turk-Browne et al., 2013; Weissman & Prado, 2012), or not (i.e., Böckler et al., 2016; Greene et al., 2009; Hietanen et al., 2006; Joseph et al., 2014; Koike et al., 2019; Lee et al., 2010; Mao et al., 2007; Noesselt et al., 2002; Peelen et al., 2004; Sato et al., 2016) of the target location. Importantly, this inclusion strategy is based on Müller et al. (2018), who recommends that a good meta-analysis might include different paradigms or tasks to investigate effects that are consistent across strategies, provided that all paradigms or tasks rely on similar higher-order supervisory control processes. Multiple experiments from the same subject group were handled according to the Turkeltaub Non-Additive procedure (HBM, 2012) to minimize the within-group effects. This together with using cluster-level thresholding allows controlling for the excessive contribution of one experiment (Eickhoff et al., 2016). For recent studies using a similar procedure see Sulpizio et al. (2020) and Langner et al. (2018).

### Activation Likelihood Estimation

Activation likelihood estimation (ALE) meta-analysis allows for assessing whether the clustering of activation foci from different experiments that tap the same cognitive function is significantly higher than that expected under the null distribution of a casual spatial association of results from the same experiments. ALE allows analysing the probability that a voxel contains at least one of the activation foci, producing a map that reflects the union of activation probabilities for each voxel. Thus, ALE assesses the overlap between foci and models the probability distributions centred on each one of them (Eickhoff et al., 2009). Using this method, we performed two separate ALE analyses on two categories of studies in relation to the type of cue (gaze vs. arrow). One investigator (CS) classified the experiments according to these categories and a second investigator (MB) double-checked the categorizations. When necessary, the three investigators discussed and resolved eventual classification doubts. After carrying out separate ALE analyses on the categories of studies, we performed a contrast analysis to directly compare the effects of the cues [(arrow > gaze) and (gaze > arrow)]. This contrast analysis allowed highlighting voxels, whose signal was greater in the first than in the second condition. We also carried out a conjunction analysis between two types of cueing [arrow ∧ gaze] to identify voxels that subtended both arrow- and gaze-cueing.

The ALE meta-analysis was performed using GingerALE 3.0.2 (brainmap.org), with MNI coordinates (Talairach coordinates were converted automatically into MNI coordinates by using GingerALE). Here we used the non-additive procedure proposed by Turkeltaub et al. (HBM, 2012) to minimize within-experiment effects. According to Eickhoff et al.’s (2009) modified procedure, the ALE values of each voxel in the brain were computed, and the null distribution of the ALE statistic was calculated for each voxel. The Full-Width Half-Maximum (FWHM) value was automatically computed because this parameter is empirically determined (Eickhoff et al., 2009). The thresholded ALE maps for the two separate ALE analyses were computed using p values from the previous step, a cluster-level inference at the 0.05 level of significance and a cluster forming threshold at p < 0.001 (uncorrected), with 1000 permutations (Eickhoff et al., 2016). The thresholded ALE maps for the contrast and conjunction analyses were computed using 10,000 permutations and a thresholding value of 0.05, with a minimum cluster size of 200 mm^3^.

## Results

### Brain Network of Gaze Cueing

**Fig. 2:**
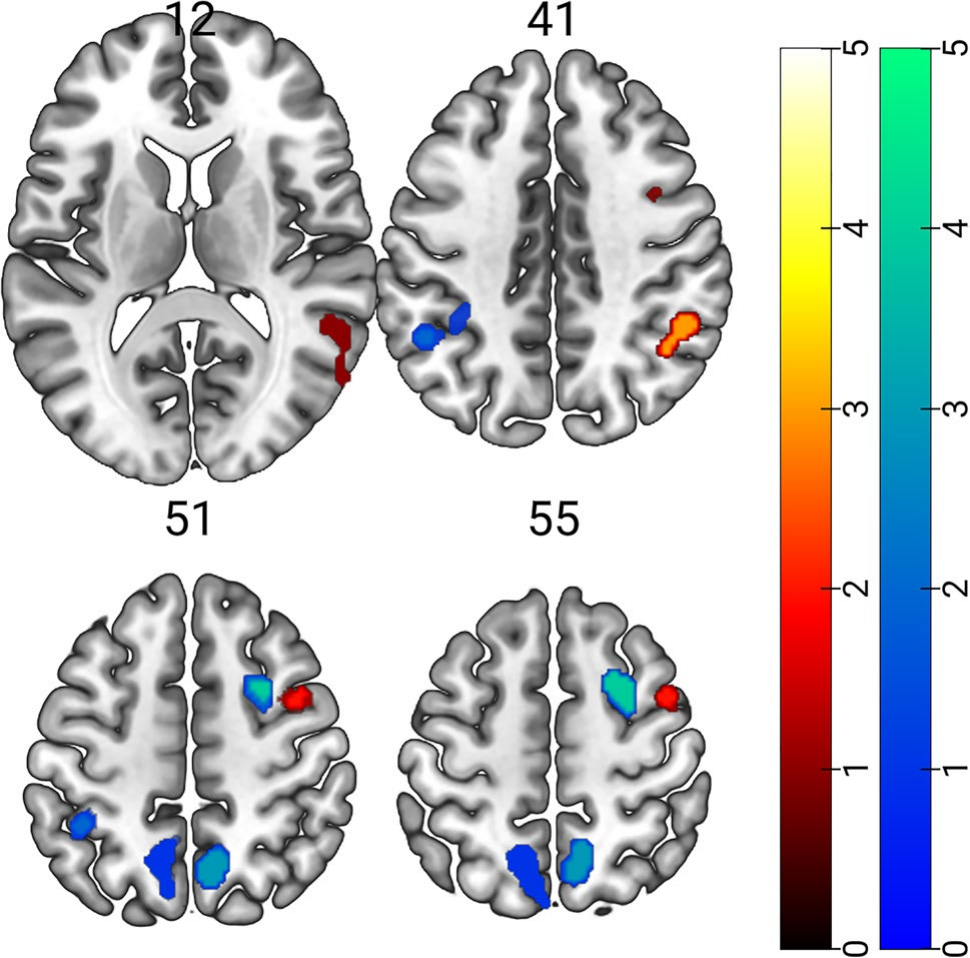
Red-to-yellow patches showed brain regions significantly activated during gaze cueing (for region labels, see Table 2); blue-to-light green patches showed brain regions significantly activated during arrow cueing (for region labels, see Table 3)

The ALE analysis on gaze cueing highlighted a cluster of activation in the middle temporal gyrus, extending towards the superior temporal gyrus and temporoparietal junction (TPJ) in the right hemisphere. This cluster encompassed the posterior portion of the superior temporal sulcus (STS). In the right hemisphere, we found activation in the precentral gyrus (PcG), extending to the middle frontal gyrus (MFG), likely corresponding to the territory of the human frontal eye fields (FEF; Amiez & Petrides, 2009)^1^. We also found a cluster of activation in the right inferior parietal lobule (IPL), extending to the angular gyrus (AG). Results are shown in Tables 2 and Fig. 2 (red-to-yellow patches).

**Table 2:** Results of the ALE meta-analysis on gaze cueing

**Cluster**	**Hem**	**Region**	**Label**	**ALE**	**P**	**Z**	**x**	**y**	**z**
1	RH	Middle Temporal Gyrus	MTG	0.019	0.000	4. 598	50	-40	6
	RH	Middle Temporal Gyrus	MTG	0.014	0.000	3.764	58	-64	14
2	RH	Precentral Gyrus	PcG	0.020	0.000	4.755	38	2	48
	RH	Middle Frontal Gyrus	MFG	0.015	0.000	4.038	42	2	54
3	RH	Inferior Parietal Lobule	IPL	0.020	0.000	4.861	50	-46	42
	RH	Angular Gyrus	AG	0.014	0.000	3.709	42	-54	40

For each cluster hemisphere, region, label, ALE value, peak p and z, and MNI coordinates are provided

### Brain Network of Arrow Cueing

The individual ALE analysis on arrow cueing revealed bilateral clusters of activation in the bilateral precuneus, extending towards the superior parietal lobule in the left hemisphere. We also found cluster of activation in the left inferior parietal lobule (IPL), mainly located in the intraparietal sulcus (IPS). The right superior frontal gyrus, in the territory of the human FEF^2^, was consistently activated for arrow cueing, as well. Results are shown in Tables 3 and Fig. 2 (blue-to-light green patches).

**Table 3:** Results of the ALE meta-analysis on arrow cueing

**Cluster**	**Hem**	**Region**	**Label**	**ALE**	**P**	**Z**	**x**	**y**	**z**
1	LH	Precuneus	pCU	0.018	0.000	4.983	-12	-60	52
	LH	Precuneus	pCU	0.015	0.000	4.465	-8	-68	56
	LH	Precuneus	pCU	0.014	0.000	4.161	-18	-54	60
	LH	Precuneus	pCU	0.009	0.000	3.303	-2	-48	48
2	LH	Inferior Parietal Lobule	IPL	0.016	0.000	4.525	-48	-50	44
	LH	Inferior Parietal Lobule	IPL	0.015	0.000	4.511	-36	-44	46
3	RH	Precuneus	pCU	0.021	0.000	5.441	8	-62	52
4	RH	Superior Frontal Gyrus	SFG	0.018	0.000	5.024	24	6	56

For each cluster hemisphere, region, label, ALE value, peak p and z, and MNI coordinates are provided

### Conjunction Analysis

The conjunction analysis revealedno suprathreshold clusters of activation.

### Contrast Analyses

#### Gaze vs. Arrow Cueing

This contrast showed no suprathreshold clusters of activation.

#### Arrow vs. Gaze Cueing

**Fig. 3:**
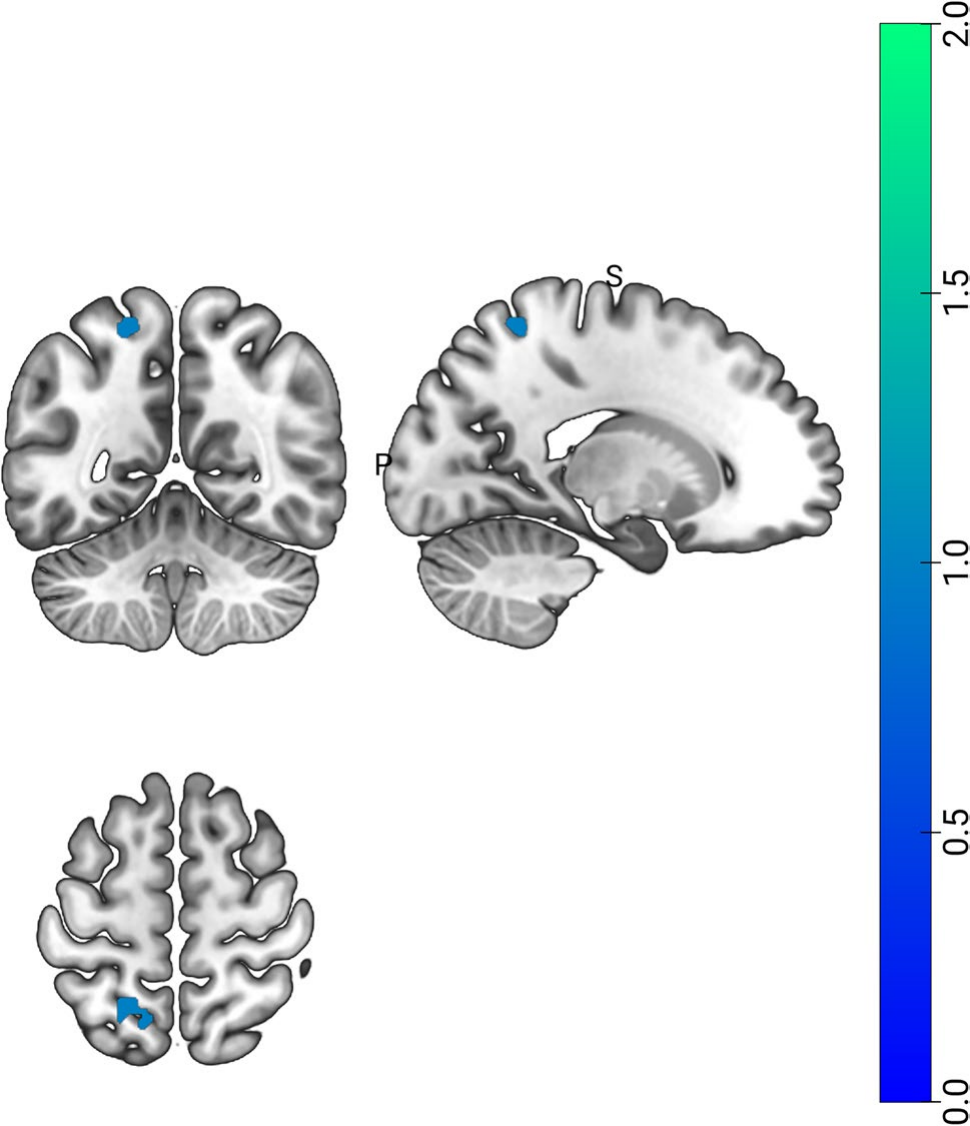
Results of the contrast analysis between arrow and gaze cueing. Blue-to-light green patches show brain regions significantly activated. For region labels, see Table 4

The direct comparison between arrow cueing and gaze cueing showed clusters of voxels spanning from the precuneus to the superior parietal lobe in the left hemisphere; these regions showed stronger convergence for experiments using arrow cueing as compared to those using gaze cueing. Results are shown in Table 4; Fig. 3.

**Table 4:** Results of the contrast analysis between arrow and gaze cueing

**Region**	**Label**	**Hem**	**P**	**Z**	**x**	**y**	**z**
Precuneus	pCU	LH	0.000	3.431	-18	-55.2	64
Superior Parietal Lobule	SPL	LH	0.000	3.352	-21.2	-57.2	60.4
Superior Parietal Lobule	SPL	LH	0.000	3.238	-11.7	-60	59.7

For each peak of the cluster region, label, hemisphere, peak p and z, and MNI coordinates are provided

## Discussion

The present ALE-meta-analysis was aimed at assessing the brain areas activated by gaze and arrow cues to shed some light on the unresolved question of whether orienting attention by social, gaze and non-social, arrow cues differ regarding the neural mechanisms involved. To date, there are two competing hypotheses: one posing that the attentional mechanisms triggered by gaze cues are different from those involved by non-social cues such as arrows, and consequently orienting by these two types of cues should rely on different neural underpinnings. Alternatively, the same attentional mechanism is triggered by gaze and arrow cues, and therefore orienting based on these cues relies on the same neural circuits. We compared the clustering of activation foci from 40 single experiments, involving a total number of 806 participants, from the 20 selected papers, assessed the overlap between foci, and modelled the probability distributions centred on each one of them (Eickhoff et al., 2009). Our findings support the claim that orienting of attention by social gaze and by non-social arrow cues are at least in part functionally segregated. Findings of the conjunction analysis did not reveal any suprathreshold clusters of activation and only social gaze cues activate brain areas involved in processing variant aspects of faces and in mental state attribution. This is in keeping with the proposal that the neural mechanisms involved in shifting attention based on social cues also rely on those involved in mental state attribution (see Calder et al., 2002; Senju et al., 2004), and with evidence showing that children orient their attention based on gaze cues earlier than they do based on arrow cues (e.g., Jakobsen et al., 2013).

More specifically, our findings show that the cluster of activation for gaze cues encompassed the posterior portion of the Superior Temporal Sulcus (STS), underlying processing variant aspects of faces such as gaze, facial expression, and lip movements (Engell & Haxby, 2007), suggesting the involvement of the temporoparietal junction, linked to mental state attribution (e.g., Schurz et al., 2017). The anterior STS is associated with gaze perception and the posterior STS with expectancy violations, such as those elicited by the incongruence between gaze direction and a salient target (e.g., Vander Wyk et al., 2009). This cluster of activity is compatible with the proposal that the temporoparietal junction reflects a link between the STS and lateral parietal regions, which mediates gaze-cued attentional shifts (i.e., Carlin & Calder, 2013). Importantly, this area is not present in the cluster of activation for arrow cues, which in turn shows bilateral frontoparietal activation, encompassing the superior frontal gyrus, precuneus, and the superior parietal lobule. The connections between the precuneus and the superior frontal gyrus have been linked to dorsal attention and frontoparietal control networks (Luo et al., 2019). In contrast, for gaze cues we found a cluster of activation in the right IPL, pointing towards a role of this region in the endogenous orienting and maintenance of attention to a target location. For both types of cues, there was a pattern of activation encompassing the territory of human frontal eye fields (FEF). Although, when contrasting the pattern of activation observed for the gaze and arrow cues, only arrow cues were associated with stronger activity in the area spanning from the precuneus to the superior parietal lobe in the left hemisphere. To our knowledge, the contribution of the FEF to social attention is not well documented and the present findings point to the FEF being involved in both social and non-social orienting (e.g., Torriero et al., 2019). Therefore, the present findings show that brain regions involved in endogenous attention – in the territory of the FEF in the superior frontal gyrus – are consistently activated by both gaze and arrow cueing. This is not surprising, as the FEF cluster of activity reflects overt attentional shifts to the cued location linked to eye movements (e.g., Schlag-Rey et al., 1997), whereas the activity of the FEF and the IPS has been linked to sustained maintenance of attention at peripheral locations (e.g., Kelley et al., 2007). However, although both arrow- and gaze-cues activated the FEF, the activation for arrow cues is more toward the superior FEF whereas that for gaze cues is toward the middle FEF. The function of this fine-grained localization is for future research to clarify. Importantly, the cluster of activation for gaze cues was lateralized to the right hemisphere is in keeping with lateralization for face processing (see Rossion & Lochy, 2022 for a recent review) and emotion-attention lateralization (e.g., De Luca et al., 2020; Pecchinenda et al., 2021; see also Hartikainen, 2021 for a recent review).

The present findings also show the activity of the right middle frontal gyrus for gaze-cues, and this area is one of the core regions of the ventral attention network that also includes the temporoparietal junction. The activity of the right middle frontal gyrus reflects reorienting attention, as it is the convergence site of the dorsal and ventral attention networks (e.g., Corbetta et al., 2008), and it is seen as a circuit-breaker to interrupt ongoing endogenous attentional processes in the dorsal network and reorient attention to an exogenous stimulus (e.g., Doricchi et al., 2010). Therefore, the right middle frontal gyrus would exert control over the dorsal and the ventral attention networks, for the flexible modulation of endogenous and exogenous attention. Within the network activation to targets, which is greater with spatial unpredictability, our findings for gaze cues point to a pivotal role of the right temporoparietal junction and the cuneus indicating engagement by stimulus-driven orienting, including activation due to target appearance at one spatial location. This evidence has been interpreted as showing two largely dissociated neural networks mediating endogenous and exogenous/stimulus-driven control of visuospatial selective attention (for a review, see Geng & Vossel, 2013), although the specific contribution of the IPS to endogenous or exogenous orienting of attention is still a matter of debate (e.g., Vandenberghe et al., 2005; Serences et al., 2005; Corbetta et al., 2008; Vandenberghe & Gillebert, 2009).

Some limitations of the current meta-analysis should be acknowledged. Firstly, the necessity to collapse valid and invalid trial data of different studies. Although this allows to achieve good power to reliably detect common areas activated during orienting of attention to social and non-social cues and it is in keeping with current recommendations (Müller et al. 2018), it precludes disentangling the neural mechanism underlying the advantages in shifting attention on valid trials from those underlying the costs of shifting attention on invalid trials for social and non-social cues. Secondly, although the method used in the current meta-analysis avoids capitalization on chance (e.g., cherry-picking the voxels showing the highest correlations), circumventing the potential risk of bias also relies on the goodness of the individual studies included in the meta-analysis. In fact, the risk of publication bias is still an open issue (see Müller et al. 2018). Moreover, coordinate-based algorithms are insensitive to non-significant results and the publication bias may go unnoticed. This is because coordinate-based neuroimaging meta-analyses are conceptually different from conventional effect-size meta-analyses as they test spatial convergence of effects across experiments with the null-hypothesis, which assumes random spatial convergence. We believe that the current results are pivotal in contributing to future research by pointing to new regions of interest into which theoretically sound investigations may be performed and by guiding fMRI studies to conduct hypothesis-driven analyses. Therefore, the current meta-analysis would have the potential impact of contributing to reducing cherry-picking. Moreover, it should also be noted that gaze and arrow cues may call upon different attentional selection mechanisms– object-based attention for gaze cueing and location-based attention for arrow cueing (i.e., Marotta et al., 2012; for more recent evidence, see Chacón-Candia et al., 2020). However, the current ALE-meta-analysis does not allow to disentangle between them. This would require using specific experimental manipulations, which may not be suitable in conjunction with fMRIs, but could be used in combination with Non-Invasive Brain Stimulation techniques. Finally, it would be interesting to extend the findings of the present meta-analysis by including experimental characteristics of the individual studies as moderators, but this requires more studies using similar methodologies.

## Conclusions

In conclusion, findings from the present ALE-meta-analysis show that orienting attention by gaze and arrow cues relies on the activation of brain regions involved in endogenous attention (e.g., the FEF in the region of the superior frontal gyrus), whereas brain regions involved in exogenous attention and mental state attribution (e.g., temporoparietal junction, middle frontal gyrus) are activated only by gaze cues. This finding points toward gaze- and arrow cues – two perceptually asymmetrical stimuli, one social and the other non-social – being overlearned signals that yield efficient attentional orienting, which is subserved by partially segregated brain networks. Albeit the present findings are correlational, they could guide future research using Non-Invasive Brain Stimulation to directly assess the contribution of brain areas involved in endogenous and exogenous attention in orienting attention by social and non-social, directional cues. Overall, the present results provide a theoretically motivated network that future neuroimaging studies, especially fMRI studies, may use to frame their analyses, in a hypothesis-driven fashion (Turkeltaub et al., 2002; Poldrack, 2007).

## Data Availability

The author confirms that all data generated or analysed during this study are included in this published article. The data that support the findings of this study are available on request from the corresponding author [AP].

## Appendix 1

Table 5

**Table 5:** PRISMA checklist (Page et al., 2021)

**Section and topic**	**Item #**	**Checklist item**	**Location where item is reported**
Title			
Title	1	Identify the report as a systematic review.	In title: ALE meta-analysis
Abstract			
Abstract	2	See the PRISMA 2020 for Abstracts checklist.	
Introduction			
Rationale	3	Describe the rationale for the review in the context of existing knowledge.	pp. 4–5
Objectives	4	Provide an explicit statement of the objective(s) or question(s) the review addresses.	p. 5
Methods			
Eligibility criteria	5	Specify the inclusion and exclusion criteria for the review and how studies were grouped for the syntheses.	pp. 5–8
Information sources	6	Specify all databases, registers, websites, organisations, reference lists and other sources searched or consulted to identify studies. Specify the date when each source was last searched or consulted.	p. 5
Search strategy	7	Present the full search strategies for all databases, registers and websites, including any filters and limits used.	p. 5
Selection process	8	Specify the methods used to decide whether a study met the inclusion criteria of the review, including how many reviewers screened each record and each report retrieved, whether they worked independently, and if applicable, details of automation tools used in the process.	p. 7
Data collection process	9	Specify the methods used to collect data from reports, including how many reviewers collected data from each report, whether they worked independently, any processes for obtaining or confirming data from study investigators, and if applicable, details of automation tools used in the process.	p. 14
Data items	10a	List and define all outcomes for which data were sought. Specify whether all results that were compatible with each outcome domain in each study were sought (e.g. for all measures, time points, analyses), and if not, the methods used to decide which results to collect.	Table 1
	10b	List and define all other variables for which data were sought (e.g. participant and intervention characteristics, funding sources). Describe any assumptions made about any missing or unclear information.	p. 14
Study risk of bias assessment	11	Specify the methods used to assess risk of bias in the included studies, including details of the tool(s) used, how many reviewers assessed each study and whether they worked independently, and if applicable, details of automation tools used in the process.	p. 21
Effect measures	12	Specify for each outcome the effect measure(s) (e.g. risk ratio, mean difference) used in the synthesis or presentation of results.	p. 15–16
Synthesis methods	13a	Describe the processes used to decide which studies were eligible for each synthesis (e.g. tabulating the study intervention characteristics and comparing against the planned groups for each synthesis (item #5)).	pp. 5–7, and Fig. 1
	13b	Describe any methods required to prepare the data for presentation or synthesis, such as handling of missing summary statistics, or data conversions.	p. 14
	13c	Describe any methods used to tabulate or visually display results of individual studies and syntheses.	p. 14
	13d	Describe any methods used to synthesize results and provide a rationale for the choice(s). If meta-analysis was performed, describe the model(s), method(s) to identify the presence and extent of statistical heterogeneity, and software package(s) used.	p. 14
	13e	Describe any methods used to explore possible causes of heterogeneity among study results (e.g. subgroup analysis, meta-regression).	None
	13f	Describe any sensitivity analyses conducted to assess robustness of the synthesized results.	None
Reporting bias assessment	14	Describe any methods used to assess risk of bias due to missing results in a synthesis (arising from reporting biases).	p. 21
Certainty assessment	15	Describe any methods used to assess certainty (or confidence) in the body of evidence for an outcome.	p. 21
Results			
Study selection	16a	Describe the results of the search and selection process, from the number of records identified in the search to the number of studies included in the review, ideally using a flow diagram.	Figure 1
	16b	Cite studies that might appear to meet the inclusion criteria, but which were excluded, and explain why they were excluded.	None
Study characteristics	17	Cite each included study and present its characteristics.	Table 1
Risk of bias in studies	18	Present assessments of risk of bias for each included study.	p. 21
Results of individual studies	19	For all outcomes, present, for each study: (a) summary statistics for each group (where appropriate) and (b) an effect estimate and its precision (e.g. confidence/credible interval), ideally using structured tables or plots.	None
Results of syntheses	20a	For each synthesis, briefly summarise the characteristics and risk of bias among contributing studies.	None
	20b	Present results of all statistical syntheses conducted. If meta-analysis was done, present for each the summary estimate and its precision (e.g. confidence/credible interval) and measures of statistical heterogeneity. If comparing groups, describe the direction of the effect.	None
	20c	Present results of all investigations of possible causes of heterogeneity among study results.	None
	20d	Present results of all sensitivity analyses conducted to assess the robustness of the synthesized results.	None
Reporting biases	21	Present assessments of risk of bias due to missing results (arising from reporting biases) for each synthesis assessed.	p. 21
Certainty of evidence	22	Present assessments of certainty (or confidence) in the body of evidence for each outcome assessed.	p. 21
Discussion			
Discussion	23a	Provide a general interpretation of the results in the context of other evidence.	pp. 19–21
	23b	Discuss any limitations of the evidence included in the review.	p. 21
	23c	Discuss any limitations of the review processes used.	p. 21
	23d	Discuss implications of the results for practice, policy, and future research.	p. 22
Other information			
Registration and protocol	24a	Provide registration information for the review, including register name and registration number, or state that the review was not registered.	In “Statements and declarations”
	24b	Indicate where the review protocol can be accessed, or state that a protocol was not prepared.	In “Statements and declarations”
	24c	Describe and explain any amendments to information provided at registration or in the protocol.	
Support	25	Describe sources of financial or non-financial support for the review, and the role of the funders or sponsors in the review.	In “Statements and declarations”
Competing interests	26	Declare any competing interests of review authors.	In “Statements and declarations”
Availability of data, code and other materials	27	Report which of the following are publicly available and where they can be found: template data collection forms; data extracted from included studies; data used for all analyses; analytic code; any other materials used in the review.	In “Statements and declarations”

## Footnotes

1 Posterior probability of the term “eye fields” to be used in a study if activation is observed at x = 38, y = 2, z = 48 is 0.85 (data from neurosynth.org; Yarkoni et al., 2011).

2 Posterior probability of the term “eye fields” to be used in a study if activation is observed at x = 24, y = 6, z = 56 is 0.80 (data from neurosynth.org; Yarkoni et al., 2011).

## Declarations

### Registration and Protocol

The present review was not registered and a protocol was not prepared.
